# Proof of concept of the Universal Baby video innovation for early child development in Lima, Peru

**DOI:** 10.1093/jpepsy/jsae035

**Published:** 2024-06-14

**Authors:** Adrianne K Nelson, Christa J Griest, Llubitza M Munoz, Nancy Rumaldo, Ann C Miller, Guadalupe M Soplapuco, Leonid Lecca, Sonya S Shin, Llalu R Acuña, Yesica V Valdivia, Alicia R Ramos, Diego G Ahumada, Blanca R H Ramos, Sarah A Mejia, Esther O Serrano, William H Castro, Victoria E Oliva, Annie S Heyman, Lauren P Hartwell, Ronnie L Blackwell, Diego F Diaz, Martha M Vibbert

**Affiliations:** Division of Global Health Equity, Brigham and Women’s Hospital, Boston, MA, United States; Boston Medical Center, Boston, MA, United States; Socios En Salud, Partners in Health, Lima, Peru; Socios En Salud, Partners in Health, Lima, Peru; Department of Global Health and Social Medicine, Harvard Medical School, Boston, MA, United States; Blavatnik Institute, Harvard Medical School, Boston, MA, United States; Socios En Salud, Partners in Health, Lima, Peru; Socios En Salud, Partners in Health, Lima, Peru; Division of Global Health Equity, Brigham and Women’s Hospital, Boston, MA, United States; Department of Global Health and Social Medicine, Harvard Medical School, Boston, MA, United States; Socios En Salud, Partners in Health, Lima, Peru; Socios En Salud, Partners in Health, Lima, Peru; Socios En Salud, Partners in Health, Lima, Peru; Socios En Salud, Partners in Health, Lima, Peru; Socios En Salud, Partners in Health, Lima, Peru; Wheelock College of Education and Human Development, Boston University, Boston, MA, United States; Socios En Salud, Partners in Health, Lima, Peru; Socios En Salud, Partners in Health, Lima, Peru; Boston Medical Center, Boston, MA, United States; Department of Psychiatry, Boston University Chobanian & Avedisian School of Medicine, Boston, MA, United States; Boston University School of Public Health, Boston, MA, United States; Wheelock College of Education and Human Development, Boston University, Boston, MA, United States; Socios En Salud, Partners in Health, Lima, Peru; Boston Medical Center, Boston, MA, United States; Department of Psychiatry, Boston University Chobanian & Avedisian School of Medicine, Boston, MA, United States

**Keywords:** early child development, community health workers, video, contingent interaction, PICCOLO

## Abstract

**Objective:**

Community-based video interventions offer an effective and potentially scalable early interaction coaching tool for caregivers living in low resource settings. We tested the Universal Baby (UB) video innovation; an early interaction coaching tool using video sourced and produced locally with early child development (ECD) expert supervision.

**Methods:**

This proof-of-concept study enrolled 40 caregivers of children ages 10–18 months assigned to intervention and control groups by health establishments in Carabayllo, Lima, Peru. Mother/child dyads received 12 weekly group health education sessions with social support. Of those, 16 caregivers also received 6 UB videos featuring brain science education and local clips of responsive, reciprocal interaction, also known as “serve and return” interaction. Survey data assessed feasibility and acceptability of the intervention. We assessed improved quality of mother/child interaction using the Parenting Interactions with Children: Checklist of Observations Linked to Outcomes (PICCOLO).

**Results:**

We found the program feasible. We successfully trained the local team to produce UB videos using locally-sourced footage and delivered the videos as part of a community-based intervention. We also found it to be acceptable in that participants enthusiastically received the UB videos, reporting they enjoyed being videotaped, and learned how to recognize and appropriately respond to their child’s nuanced sounds and gestures. The median change in total PICCOLO scores favored the intervention group compared to the control group.

**Conclusions:**

UB offers great potential as a sustainable, potentially scalable, and culturally appropriate tool to promote equity for child development among young children living in low resource homes globally.

Lancet’s 2016 series *Advancing Early Childhood Development: from Science to Scale* estimates that 250 million children under five are at risk of failing to attain their full developmental potential (Lu et al., 2016). These children need healthy nutrition, freedom from disease, a safe environment, opportunities to learn and explore, and stimulating caregiver–child interaction to thrive (Daelmans et al., 2015; Horton, 2017; Uchitel et al., 2019).

Negative early experiences impede healthy neurodevelopment (Benjet et al., 2010; Schilling et al., 2008; Shonkoff et al., 2012) and can lead to delay and slow school advancement (Luby et al., 2013), problems with mental health and relationships (Benjet et al., 2010), and early onset of chronic disease (Sonu et al., 2019). Responsive caregiving between a caregiver and young child buffer these consequences by increasing neural brain activity related to cognitive processing, white matter connectivity, regulation of the stress response systems, synaptic pathways, and brain volume (Hertzman & Boyce, 2010; Stiles, 2008; Walker et al., 2011, 2007). In countries where poverty, poor nutrition and disease pose additional threats to children’s cognitive development, positive stimulation through parent–infant interactions is a sustainable protective factor (Blair & Raver, 2016; Brown et al., 2017; Horton, 2017; Luby et al., 2013; Morris et al., 2017).

Early interventions that encourage and coach caregivers to stimulate their child’s development show efficacy (Andrew et al., 2020; Grantham-McGregor & Smith, 2016; Obradovic et al., 2016). Interventions that promote caregivers’ responses to young children’s attention, vocalizations, play, and exploration are particularly effective (Jeong et al., 2021; Morris et al., 2017), yet sparsely provided in resource-limited areas in part due to difficulty scaling up effective programs (Bornstein et al., 2012; Bornstein & Putnick, 2012; Lansford et al., 2022). In order to scale parenting programs for efficient, equitable, and cost-effective dissemination, researchers must address challenges of scale and specificity.

To address these challenges, we examine video as a tool for parent coaching. Video offers feasible, efficient, economical, and equitable program access for many families coping with barriers common to low resource settings (Alvarenga et al., 2020; Feil et al., 2020). Audiovisual demonstrations are especially effective at capturing people’s attention, engaging emotions, and enhancing the learning of new behaviors (Webster-Stratton, 1982, Webster-Stratton et al., 1988), and promote peer role models who can influence attitudes, beliefs, motivations, and behaviors (Columbo, 2001; Magill-Evans et al., 2007; Murphy, 2001).

The use and evaluation of video *viewing* interventions for parent coaching in low- and middle-income countries is relatively new. Most video coaching interventions have been developed and studied in high-income countries, and most rely on individual *feedback to a caregiver usually by a clinician* (Cates et al., 2018; Stegenga et al., 2018). These and other video feedback approaches have shown the power of visual input to influence caregiver learning, in the context of a clinician who guides and mediates a caregiver’s viewing experience. One trial in Salvador, Brazil found that mothers receiving an 8-week video feedback intervention interpreted meaning of their infants’ behavior more often, asked more questions, and were less intrusive compared to controls (Alvarenga et al., 2020). A meta-analysis conducted on 26 individual video feedback interventions by Fukkink et al. in 2008 found average effect sizes on parenting behaviors and small to average on child development (Fukkink, 2008). However, despite their power to influence caregiver knowledge and child outcomes, video *feedback* interventions are limited in their potential for scalability because they require the mediation of an expert provider and individual time with each caregiver. In contrast, video *viewing* interventions, if feasible and effective, will likely have far more promise for reaching large populations, especially in low- and middle-income settings where staffing and resources are scarce. We found no evidence of interventions in low-income countries that test the impact of combined video viewing *alone* (*without individual feedback*) within group health education sessions.

We combined an innovative video-based approach—*Universal Baby (UB)—*with community-based parent health education sessions. UB consists of structured short videos that are co-created with local families to deliver visually rich information about early brain development and community-specific, culturally tailored messaging and behavioral modeling. The objective of UB is to coach caregivers to take part in reciprocal and contingent (“serve and return”) patterns of interaction during daily activities with their child. We designed a proof of concept study to determine whether UB has potential, and merits testing in a larger randomized, pilot design (Hilliard et al., 2021). As part of this proof of concept study, we investigated the feasibility (whether the concept can realistically be implemented) and acceptability (the population’s interest and approval of the UB video intervention) within the context of a community-based early intervention (Hilliard et al., 2021). We also present exploratory findings on mother/child interactions.

## Methods

### Study site

This study took place in the district of Carabayllo, Peru, located north of the Lima metropolitan area. Carabayllo is one of the largest and oldest districts in the city and is home to over 330,000 people, 19.2% of whom live in poverty (Ministerio de Desarollo e Inclusión Social, 2023). Carabayllo has approximately 8,800 children under 2 years of age (Pessah et al., 2018), 70% of whom are at risk for delay, based on prior work (Muñoz et al., 2017).

Carabayllo was chosen because it is the founding site of the nongovernmental organization Partners In Health (SES—Socios En Salud, Peru) and the base for the study team’s work in early child development (ECD) since 2012.

### Universal Baby: Intervention description

#### Theoretical underpinnings and formative work

Dr Martha Vibbert developed UB 10 years ago with authors CG and ATK (*Unviersal Baby Project*, 2023). UB is grounded in social learning theory, which views learning as a cognitive process shaped by individual experience, behavioral modeling, and environmental factors (Bandura, 1977; Cherry, 2021). Each video incorporates mediational processes of observational learning including attention, retention, reproduction, and motivation. UB combines messages to reinforce: (a) social norming within a local context, (b) clear visual information to provide a cognitive understanding of how a caregiver’s responsive behaviors impact a child’s development, and (c) motivation to intentionally practice behaviors.

Many parenting video approaches rely on studio-based (i.e., staged), literacy-dependent, and overly generic guidance (“play with your baby”) biased to Western, middle- and upper-class childrearing routines. UB addresses these limitations by centering culturally authentic, locally sourced video footage of parents engaged in observable (and often nuanced) everyday interactions with their young child during naturally occurring activities in local caregivers’ homes. UB videos honor local traditions while also presenting accurate brain science with compelling visuals that link a child’s internal brain processes to “serve and return” interaction and neurodevelopmental outcomes.

#### Core UB content and structure

**Figure 1.**
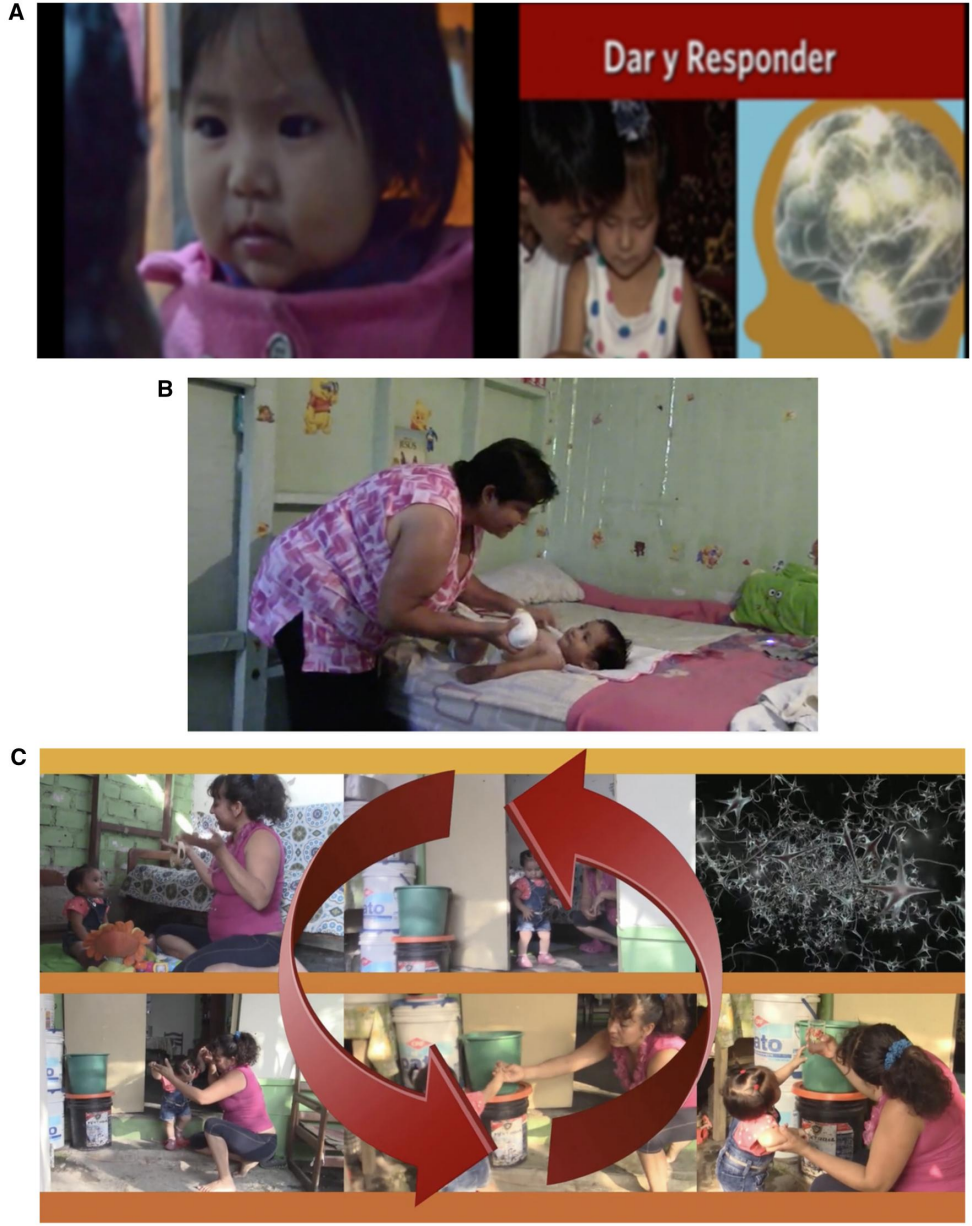
(A–C) Sample Universal Baby video montage, Carabayllo, Peru. (A) Opening footage. Videos open with “establishing shots” of caregivers from across the globe engaging in interaction exchanges with their children, as well as imagery of brain development phenomena related to responsive interactions. (B) Locally-sourced caregiver–child interaction. Imagery of caregiver–child interaction allowing for close, sequential observation of naturalistic responsive caregiving. (C) Slow motion emphasis of caregiver–child interaction and relationship to synaptic proliferation in the brain. Key moments of the interaction are emphasized through slow motion, narration, visual repetition, and graphics such as moving arrows and pop-out font. These visual clips also highlight the sequence and subtle nuances that often accompany responsive interaction exchanges. We received permission for the use of all copyrighted images. Credits from left to right: [Image A (child): Universal Baby, with Socios En Salud, Image A (father and child): World Health Organization (with UNICEF), Image A (brain imaging) Harvard Center on the Developing Child].

Figure 1(A–C) illustrates some of the core elements common to UB videos. Videos open with “establishing shots” of caregivers engaging with their children using imagery of local parents and community scenery. This imagery is intercut with interaction images of caregivers and children from elsewhere in order to encourage the viewer to connect with an international community of caregivers while also identifying with caregivers in their own community. Additional visual sequences and narration highlight internal structures and regions in a child’s brain, synaptic connections being formed, and neuronal density as these relate to caregiver–child interactions and later child development outcomes.

Then, UB videos zero in on several minutes of locally-sourced caregiver–child interaction, that allow for close, sequential observation of naturalistic responsive caregiving. These interaction segments demonstrate, in real time, how “serve and return” exchange moments are initiated via child signals, how they unfold spontaneously, and how they are sustained by ongoing reciprocal responses by caregiver and child. Key moments of the interaction are emphasized through slow motion, narration, visual repetition, and graphics. These clips highlight the sequence and subtle nuances that accompany responsive interaction exchanges. Video footage is closely vetted by local partners, and script is co-developed with partner teams based on previously field-tested templates adapted to local language vernacular. Colorful local imagery and music are added to enhance viewers’ engagement and community recognition.

We co-created six UB videos with local volunteer caregivers and their child (ages 6–24 months) identified via existing social networks and word of mouth. Volunteers were a separate group from those enrolled in the study described below. All videotaped caregivers completed an in-depth informed consent process and an assent on their child’s behalf. Each dyad received a food basket of approximately $10 in value. Volunteers participated in a focus group about their experience.

#### Group health education sessions

A locally trained community health worker (CHW) delivered group health education sessions to all enrolled mother/child dyads at cultural centers and health clinics. We designed the sessions based on the Ministry of Health’s Strategies for Health Promotion. Activities included songs to engage children, and basic health education topics like hygiene, infectious disease prevention, domestic violence prevention, parenting, and maternal depression. CHWs facilitated loosely structured social support where caregivers were encouraged to share freely and respond to one another. In the UB intervention arm, the group viewed bi-weekly UB videos after group sessions.

### Study design

This is not an efficacy trial and was not powered to measure significant impact on study outcomes (Czajkowski et al., 2015). We sought to explore the impact of UB video content when delivered with group sessions, to inform a future effectiveness trial. Therefore, we designed a nonrandomized prospective study with group allocation at the health center level. The convenience sample of 40 dyads was evenly divided by allocation. Between April 2015 and April 2016 all participants received 12 weekly health education group sessions. We evaluated results using video coding, self-report, and observation tools at enrollment (baseline) and after the completion of the intervention (postintervention).

### Recruitment and enrollment

We chose three Carabayllo clinics using a random drawing of all clinics in the district. After the selection, the team assigned two small health centers to the intervention group and one large enough to accommodate all control participants to the control group.

Eligibility criteria included being the primary caregiver of a child aged 10–18 months “at risk” or “delayed” in at least one area of the four domains of the psychomotor development assessment instrument endorsed by the Peruvian Ministry of Health: the Escala de Evaluación del Desarollo Psicomotor, EEDP (Rodriguez, 1996). Children with any known medical condition that would make them unresponsive to early intervention, those >21 days premature, and those who were not at risk per the EEDP (M Schreiner, 2008) were excluded. The team identified children through community-based active case finding and clinician referral. We invited all eligible dyads to participate on a first-come, first-serve basis, reviewed the details of the study with them, and requested the caregiver sign an informed consent.

#### Sociodemographic measures

We collected demographic, socioeconomic status, and maternal depression data. We measured poverty using the Progress Out of Poverty Index (Schreiner, 2008) and maternal depression using the Hopkins Symptoms Checklist (Derogatis et al., 1974), both which we have used in previous ECD studies in this setting (Miller et al., 2021; Nelson et al., 2018). Trained SES staff recorded data in both paper and digital formats. Staff automatically uploaded digital data into the SES information system, a password-controlled internal database with built-in quality control mechanisms.

#### Feasibility measures

**Figure 2.**
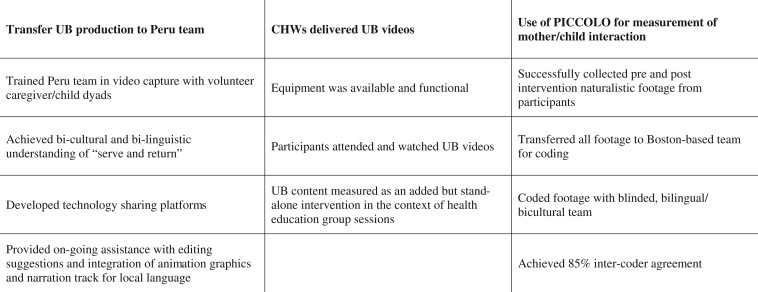
Core feasibility elements of UB

Core feasibility and acceptability measures are presented in Figure 2. We determined the study feasible if: (1) we successfully transferred production of UB videos to the Peru production team, (2) CHWs successfully delivered UB videos to the target audience as part of a broader parent/child intervention, and (3) we were able to use the PICCOLO instrument to assess the impact of UB on mother/child interaction. We developed four criteria for success for each of these goals (Figure 2).

#### Acceptability measures

We defined acceptability as follows: (1) the UB videos are acceptable from the perspective of the video volunteers who were filmed, and (2) the UB videos are acceptable according to the women who viewed them.

To learn about acceptability from the video volunteers, we developed a semi-structured survey with forced choice and open-ended questions. We asked, for instance, “what could the team do to make the [videotaping] process more comfortable?” To further examine community acceptability of being videotaped, we included all participants who were videotaped for the PICCOLO.

Mothers who received the UB intervention responded to another set of semi-structured survey questions about their experience watching UB videos. For instance, “how did you feel about watching the videos of other caregivers?”. We asked about their opinions of the video content, “what did you like/not like about the videos of other caregivers?”. Finally, we asked, “would you like to be featured in a future UB video?”.

#### Preliminary impact measures

In order to understand whether caregivers placed more importance on child development after viewing UB videos, the study team designed a questionnaire using a 4-point Likert scale. We asked caregivers to rate the importance of each area of their child’s health, including learning & education, physical health, social skills, interactions with adults, nutrition, happiness, obedience, and the child’s brain development. We then asked participants to rank these areas of their child’s health from most important to least important at baseline and postintervention timepoints using forced choice response.

We were not powered to assess impact, however, in order to assess potential impact for a future effectiveness trial, we measured the quality and frequency of caregiver–child interactions at baseline and within 1 month of the last 12-week session using the Parenting Interactions with Children: Checklist of Observations Linked to Outcomes (PICCOLO) instrument (Roggman et al., 2013; Vilaseca et al., 2019). The PICCOLO uses detailed coding of raw naturalistic footage to comprehensively assess caregiver–child interaction behaviors in four “developmental domains of parenting” shown to predict better child development outcomes: affection, responsiveness, encouragement, and teaching. The rating system is based on 0-2 scale, defined by frequency of observed behavior: 0 (*no behavior observed*), 1 (*emerging/rarely observed*) and 2 (*strongly, frequently observed*) (Roggman, 2009).

### Data analysis

For qualitative analyses, we chose thematic analysis because our qualitative data involve an applied health intervention, we are interested in comparing and contrasting participant perspectives, and its clear and organized approach fitted our semi-structured interview tool (Nowell et al., 2017). First, we saved open-ended survey responses in an excel file. A group of three, two students and a qualitative researcher, then identified emergent themes using thematic analysis. We created a codebook, coded qualitative data using open coding and assigned excerpts to codes. We then wrote short paragraphs for each code and wove them into the narrative. After creating a narrative, we shortened the content to focus on the acceptability of the UB intervention as presented here (Clarke et al., 2019). Excerpts were chosen based on their relevance to the study objective and their illustrative quality. We also included contrary, unusual, or surprising perspectives. We replaced participant numeric identifiers with pseudonyms for this report.

We analyzed quantitative data using Stata 15 (College Park, TX). We calculated frequency distributions in PICCOLO score change from baseline to 3-month follow-up between the two study arms, and measured the association between intervention status and PICCOLO score change using linear regression, adjusting for baseline covariates that significantly differed (*p* < .05) between intervention and control arms.

### Ethical considerations

This study was approved by the Ethics Review Board at Harvard Medical School, Boston University, and the Instituto Nacional de Salud (National Institute of Health) in Peru. Grand Challenges Canada provided seed funding for this study.

## Results

### Study participants

**Figure 3.**
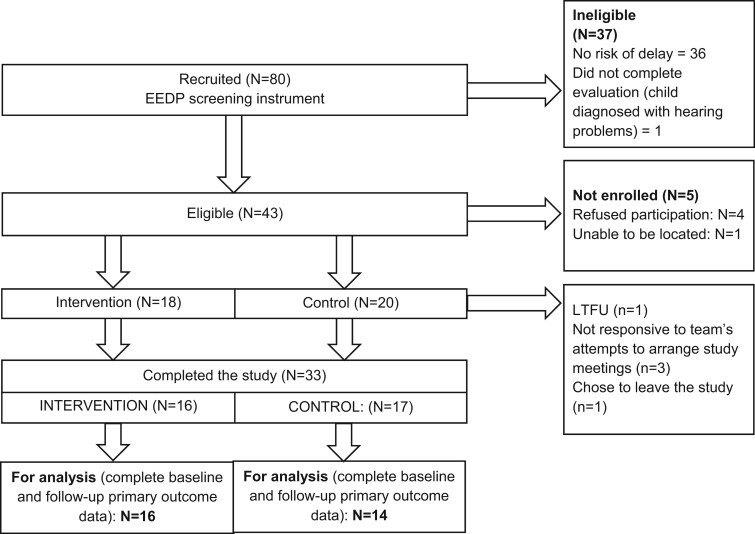
Study enrollment flow diagram.

We screened 80 children for eligibility; 43 mother–-child dyads from the cohort met eligibility criteria (Figure 3). Of those, four mothers declined participation and one could not be located. The remaining 38 were enrolled into the study (18 interventions, 20 controls). Among intervention participants, one was lost to follow-up and another chose to leave (*n* = 16 analyzed). Among control participants, three were lost to follow-up (*n* = 17 analyzed). Among the remaining 33 dyads, 30 dyads had complete PICCOLO data at baseline and postintervention.

Table 1 shows baseline characteristics of the study population. Mean child age at study initiation was 14.2 months and 60% were female. Primary caregivers were exclusively female and the child’s mother. We did not request any racial or ethnic data. Caregivers in the control arm had a higher proportion of education past secondary school (35.7% vs. 6.25%).

**Table 1.** Baseline characteristics, *N* = 30.

Child characteristics	*N* (%) or mean [SD]	Intervention arm, *n* = 16	Control arm, *n* = 14
Female	18 (60.0)	8 (50%)	10 (71.4%)
Number of weeks gestation at birth	39.0 [0.99]	39.3 [0.93]	38.7 [1.05]
Age in months	14.2 [2.41]	14.4 [2.31]	13.9 [2.58]
Ever breast fed	27 (90.0)	14 (87.5%)	13 (92.9%)
Two or more primary caregivers	5 (16.7%)	3 (18.7%)	2 (14.3%)
Self-reported alcohol during pregnancy	0	0	0
Family history of developmental delay	16 (4533%)	10 (62.5%)	6 (42.8%)
Baseline EASQ z score, vs. World Bank norms mean [SD]	−0.34 [0.81]	−0.32 [0.94]	−0.35 [0.70]
Baseline total PICCOLO median [IQR]	27.5 [18–42]	26.0 [16.5–45]	30.5 [19–42]

**Caregiver characteristics**	***N* (%) or mean [SD]**	**Intervention arm, *n* = 16**	**Control arm, *n* = 14**

Female	30 (100.0)	16 (53.3)	14 (46.7)
Mean age	26.6 [5.6]	26.6 [6.1]	26.5 [5.3]
Married or living together	25 (83.3)	14 (87.5)	11 (78.6)
Education			
Primary or less	4 (13.3)	4 (25.0)	0
Some secondary	20 (66.7)	11 (68.8)	9 (64.3)
Any technical or university	6 (20.0)	1 (6.25)	5 (35.7)
Identifies as Amerindian	27 (90.0)	15 (93.7)	12 (85.7)
No history of depression	26 (86.7)	3 (18.75)	1 (7.1)
Employed outside of household	2 (6.7)	2 (12.5)	0 (0)

## Outcomes

### Feasibility

#### Transfer of UB production to Peru team

##### Trained Peru team in video capture

Boston-based collaborators successfully trained the SES team to collect naturalistic footage from caregiver–child dyads interacting in their homes and piloted with two volunteer families. The SES team collected video when the parent and child were together and alert and brainstormed ways to encourage mothers to take part in their typical daily activities, suggesting she should “do what you would normally be doing at this time”. Some mothers expected the video team to tell her what to do or to “play” with her child during the videotaping. Mothers typically fed, bathed, cooked, and/or cleaned with her child during video sessions. The videographer collected footage with a small, unobtrusive camera and minimized intrusions into the mother/child’s interactions by staying out of view and maintaining a “non-responsive” stance.

##### Achieved bi-cultural and bi-linguistic understanding of “serve and return”

The Peru and Boston-based teams worked in close coordination throughout the entire six-video production phase, to make sure that visual imagery and script translations were vetted for accuracy and fidelity to core messages. Cultural adaptations were made collaboratively. For example, the Boston-based, SES, and CHW teams engaged in extended conversations to translate the term “serve and return” in a culturally and linguistically appropriate way. The CHW’s, SES team, and Boston-based team agreed on “dar y responder” because it was easy to remember and implies ongoing back and forth engagement. Locally composed music and images of the district were added to enhance cultural specificity and identification.

##### Developed technology sharing platforms

The SES team successfully transferred all digital video files to the Boston-based team via WeTransfer, a secure cloud-based storage and transfer platform. The SES team ensured all video files were password-protected and encrypted, both for transit across networks (TLS) as well as in storage (AES-256). Access links expired 7 days after transfer.

##### Provided ongoing assistance with video capture and production

During production, the Boston UB team traveled to Peru twice to guide SES staff on video production, shifting greater responsibility to SES staff each time. For the first video, Boston-based film editors guided video capture directions, animation details, and actual editing using AVID technology. By the second video, the SES team led the process; subsequently producing and editing the final five videos using Adobe Premier CC2014. Upon culmination of the project, the SES staff collected high quality, naturalistic footage in the field, picked key “serve-and-return” moments for use, edited footage into didactic excerpts while preserving the brain science, and produced sharable videos of 8–12 min each. Since this project, the SES team successfully trained another team at SES who produced three nutrition related UB videos.

#### CHWs delivered UB videos

##### Equipment was available and functional

CHWs were able to access videos via a USB stick, which the Peru team prepared on the computers at their office and show them on a television available at the location of the group sessions.

##### Participants attended and watched UB videos

Mother–child dyads received the health education sessions in four groups of 8–10. Mothers in the intervention group watched the six UB videos over the course of 3 months (one video every 2 weeks) as a stand-alone activity. In the case that a mother missed a session, she saw the video privately at the following session. None of the mothers who finished the sessions missed any UB videos.

##### UB content measured as a stand-alone intervention

CHWs were trained to avoid any child development teaching during sessions to prevent confounding the impact of the videos. For example, CHWs praised caregivers and children, but did not direct them to child-led or interactive play, or correct behavior that was not developmentally appropriate, unless dangerous to the child. CHWs also confirmed that they did not discuss UB videos with caregivers after delivery.

##### Use of PICCOLO for measurement of mother/child interaction

We collected nearly 23 hr of PICCOLO video interaction data via hand-held high-definition cameras and digitally uploaded them to secure SES computers. Per PICCOLO criteria, we discarded the first 5 min of each clip and used the next 10 min for coding.

The UB team scored in consultation with the instrument’s designer, Lori Roggman. Boston-based native Spanish speakers trained in early education and psychology (SM) and (DA) conducted the coding blind to study arm. CGN and MV mediated inter-coder discussions.

During three, 6-hr training sessions, coders scored 18 practice videos (10 from the User Guide, 8 from Carabayllo) independently and compared findings. By training end, the coding team successfully achieved 75% interrater reliability utilizing the expanded item-level scoring descriptions provided in the PICCOLO User Guide and an internal set of “rules”. Then, coders followed recommendations by Roggman et al. and coded two video clips per hour, 2 hr at a time, per day, to prevent fatigue. The team watched each 10-min clip of raw participant footage once through without stopping, and scored it during the second viewing, spending approximately 25 min on each.

The Boston-based team successfully conducted coder “calibration” meetings at a 20% rate (every 6 videos) during coding to prevent drift. When the coding team encountered an unfamiliar colloquial expression or gesture, the UB team cross-checked with the SES team, maintaining participant anonymity. During a final series of meetings, the coders and mediators discussed discrepancies and resolved each instance to create one final database.

### Acceptability

#### Volunteer caregivers being videotaped

Overall, caregivers (*n* = 38) expressed pride in their child during videotaping and enjoyed seeing their child on video. For most participants it was the first time they were videotaped interacting with their child. They were happy to receive a copy of the video, saying it would be very meaningful to see in the future and could help other mothers learn to interact with their children.

Several participants mentioned they were able to perceive reactions from their child in the video that they had missed during the interaction and identify moments they could have reacted differently. “See how I interact and am with my children. [In the video] I see what I am doing wrong and can improve on.” (Yesica, 26 years old)

When asked whether their activities were typical of daily activities, 100% of the women responded positively.

Of 38 participants, 27 (71.1%) would like to share the video with family members to show them that they were patient, loving, or busy with their children at home, suggesting videos boosted pride in their caregiving role. Almost all (35/38, 92.1%) said they would share the video with other caregivers. Several want to share the video to help others: “so they are a little more loving with their children” (Estela, 24 years old), and “because we don’t realize what we are doing with our children. With the video we can see and learn” (Serena, 28 years old). This indicates mothers gained valuable insight and felt comfortable enough with the representation of their culture to appropriate the content and share within their social networks.

Most participants said they felt uncomfortable when video capture began 28/38 (73.7%), but 31/38 (81.6%) said they became more comfortable. Those that expressed discomfort mentioned embarrassment about their house (3/38, 7.9%), the length of the visit (2/38, 5.3%), the number of visitors (1/38, 2.6%), or discomfort with a male visitor (1/38, 2.6%). One caregiver worried a criminal could use the video to hurt her child (1/38, 2.6%).

Four participants shared ideas for improved video capture, including planning ahead, appearing at the home without warning, self-video capture, and having a fixed camera without a videographer. Three participants suggested requesting permission to use the video following the capture rather than in advance (3/38, 7.9%). Findings suggest high variability in video capture preferences.

#### Caregivers watching UB videos

Mothers in the UB intervention group (*n* = 16) spoke extensively about skills they learned from the videos. They acquired awareness of “serve and return” interactions and said content helped them identify the child’s subtle signals, realize the importance of interaction, and acquire knowledge about children’s brain development.

Mothers were moved by video interactions and identified with the caregivers shown. This suggests the videos accurately captured daily activities and interactions widely shared in this context: *“*The mother who was bathing her child, my son does the same thing when he doesn’t want to get out, he also splashes.” (Claudia, 22 years old).

They particularly appreciated the scientific part of the video with the image of the child’s brain. They were impressed at how much growth occurred at such a young age.

“I liked more where they talked about the development of child’s brain. At 3 years old their brain is developing.” (Olivia, 23 years old).

All but one mother (15/16, 93.8%) reported they learned something new by watching the videos. They highlighted messages about the importance of interacting with their children. “It’s important to interact with our children during their first years of life, without knowing we have to understand what they need.” (Julieta, 19 years old).

Mothers spoke eloquently about being more attuned to their child after seeing the videos. They were enthusiastic about their new ability to interpret subtle signals from their child’s nonverbal behaviors and vocalizations: “I felt good because I learned new things, I saw how the mothers interacted with their children and I learned how to understand my son without him being able to tell me.” (Julieta, 19 years old).

Mothers repeatedly emphasized patience as a caregiver quality they admired in others and felt more equipped to embody after seeing the videos: “We have to be patient. Their development is important and [so is] what they capture when they’re children.” (Maria, 21 years old).

The mother’s focus on patience may demonstrate greater awareness of the child’s needs. Several mothers referenced a video in which a grandmother cares for the baby while the mother is occupied as exemplifying caregiver patience:

Because in that moment the grandma understood him, she had the patience despite him throwing a tantrum. I didn’t do it with my son, I didn’t play with him, when I fed him I thought that he should take everything now, that I have to feed him from 10 to 12 spoonfuls. Before, I insisted a lot and he would throw up. The grandma didn’t insist and now I don’t do it. (Maria, 21 years old)

All mothers said they would benefit from watching videos of themselves and videos created with footage from other mothers. They were interested in sharing videos (13/16, 81.3%, TV being the most popular way to share videos). They also emphasized the benefits of discussing the videos among peers in a group format.

#### Exploratory findings on UB impact

Overall, caregivers from the intervention group rated developmental areas of higher priority postintervention compared to preintervention, except “interaction with adults”. This was not the case with the control group, who rated only “learning and education” and “physical health” higher postintervention. With the exception of “interaction with adults”, the intervention group rated all areas higher than the control group postintervention.

When asked to rate areas of their child’s health from most important to least important, two mothers in the intervention group rated brain development as the most important area of their child’s health preintervention compared to nine postintervention. Only one rated brain development as the most important area in the control group postintervention.

**Figure 4.**
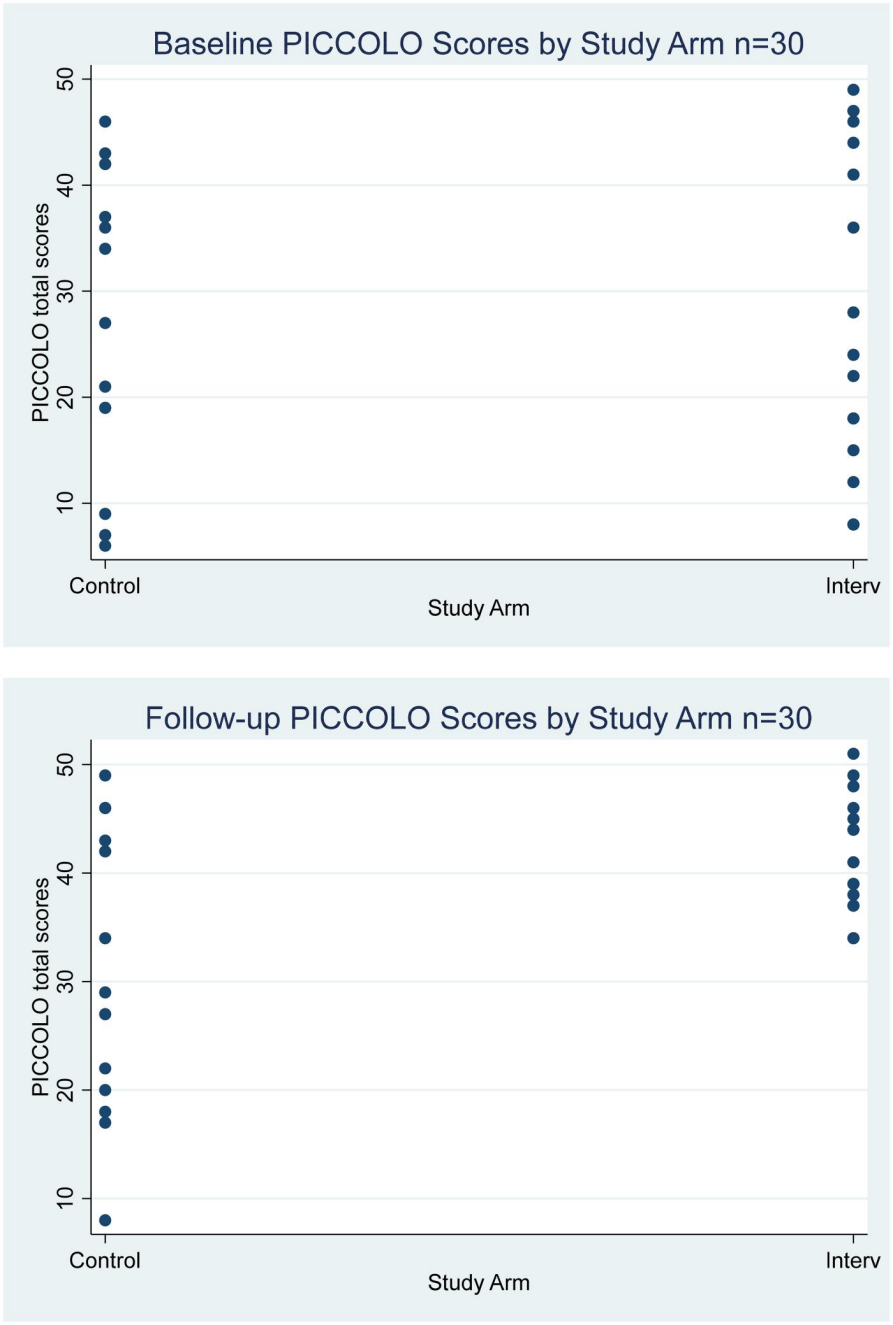
Distribution of parent outcome scores (PICCOLO) at baseline and follow-up, *N* = 30.

PICCOLO scores at baseline between study arms were similar (26 in the intervention arm and 30.5 in control arm) (Table 1), and more widely spread at follow-up (intervention total median score 41.0, control total median score 31.5) (Figure 4). The intervention arm improved their mean score significantly more than the control arm in adjusted analysis (beta 13.6, 95%CI 0.2 to 27.4, *p* = .05, *R*^2^ = 0.14, *F*(2,27) = 2.21) (Supplemental Table S1).

### Discussion

We found that UB was feasible in that the video production was successfully transferred to a local team and delivered to parents using locally available technology via CHW-led group health education sessions. The PICCOLO was a feasible instrument to measure parent–child interactions through naturalistic video footage and scored by a cross-cultural team. We learned that significant attention invested in exchange around UB video script and PICCOLO coding was necessary for a successful outcome. Since this study was undertaken, the User Guide has been finalized in Spanish, meaning the local team can now conduct on-site scoring.

UB was acceptable, inspiring enthusiastic engagement among caregivers. Both video volunteers and intervention group participants found footage of local community members empowering, relatable, and effective in conveying messages about ECD and healthy parenting. They expressed interest in capturing their own parenting interactions through similar footage. Investigators collecting video footage may find providing participants with capture options increases participant comfort.

PICCOLO findings support future randomized assessment with a sample size powered to detect statistically significant improvements in mother/child outcomes, including UB’s impact on individual PICCOLO sub-domains. Should mother/child interaction improve in piloting, we could further explore UB’s impact on ECD outcomes.

UB carries advantages over current video interventions. Childrearing practices and norms for interaction are known to vary widely across cultures and contexts (Bornstein et al., 2015). Nonetheless, early interaction “exchanges” across most cultures share some basic commonalities: they rely on infant cues interpreted by a caregiver, and they evolve into sustained moments of shared focus, or bouts of “joint attention” to a third focal point (Bard et al., 2022). These bouts of joint engagement play a key role in helping the young brain make and proliferate neuronal connections. UB uses caregivers as peer experts and our data show that local examples of joint engagement in peer dyads make accessible content that participants can easily mirror in their own lives.

In contrast to video-feedback interventions, UB is delivered at low cost with trained CHWs. High-quality educational content and explicit dyadic interactions are shared to reach large caregiver and/or trainee audiences without specialist involvement. Delivery options for UB videos include existing public-health interventions, clinical waiting rooms, community-based group meetings, and broad public health TV or social media messaging. In addition to this project and further efforts in Peru, UB has been developed locally to reach viewers in diverse communities with scarce health care infrastructure, such as Uganda, Cherokee Nation, Zambia, South Africa and clinically oriented parenting programs in the USA.

#### Limitations

We had some study dropout (15%) and missing PICCOLO data due to file corruption (*n* = 3). Enrolled children who did not complete the study could be more vulnerable to the ill effects of developmental delay. However, as most dropouts were from the control arm, their inclusion would likely increase the effect.

## Conclusion

This study suggests that the innovative approach of UB—using local naturalistic video footage to co-create structured short videos grounded in a theoretical framework—is feasible, acceptable, and may increase uptake of responsive parent–child interactions. Future research will test the effectiveness of this UB intervention embedded within a community-based group health education session as part of a randomized pilot study. Proof of concept studies such as this should also investigate the cross-cultural import of UB as a potential scalable tool delivered through social media or television to achieve global equity in advancing healthy child development.

## Supplementary Material

jsae035_Supplementary_Data

## Data Availability

Data are available upon request.
