# Supplementary material for: Proof of concept of the Universal Baby video innovation for early child development in Lima, Peru
Source: J Pediatr Psychol. 2024 Jun 14;50(1):51–62. doi: 10.1093/jpepsy/jsae035 (PMC11753875; doi:10.1093/jpepsy/jsae035)
Supplement: jsae035_Supplementary_Data [file jsae035_supplementary_data.docx]

**Supplemental Table 1. Parent outcome scores (PICCOLO) by study arm at 3-month follow-up and regression model of change, N=30**

| **N=30** | **Intervention n=16** | **Control n=14** |  |
| --- | --- | --- | --- |
|  | **MEDIAN[IQR]** | **MEDIAN[IQR]** | **p value** |
| **Total** | 41.0 [37.5-44.5] | 31.5 [20.0-43.0] | 0.06 |
| **Affection** | 10.0 [8.5-12.0] | 8.0 [5.0-10.0] | **0.03** |
| **Responsiveness** | 13.0 [12.0-14.0] | 11.5 [5.0-14.0] | 0.08 |
| **Encouragement** | 10.5 [8.5-11.0] | 6.5 [4.0-11.0] | 0.07 |
| **Teaching** | 8.5 [6.0-11.0] | 7.0 [5.0-8.0] | 0.21 |
| **Change** | 14.0 [1.5-21.0] | 3.0 [-7.0-19.0] | 0.23 |
| **Regression*** | **Regression Coefficient** | **95% CI** | **p value** |
| **PICCOLO scores** | 13.56 | -0.25, 27.4 | 0.05 |
| **Maternal education above primary school** | 8.70 | -3.31, 20.72 | 0.15 |
| **Constant** | -25.29 |  |  |
| **R^2^** | 0.14 |  |  |
| **F (2,27)** | 2.21 |  |  |

*Adjusted for maternal education level
